# Electric Field Cycling of Physisorbed Antibodies Reduces Biolayer Polarization Dispersion

**DOI:** 10.1002/advs.202412347

**Published:** 2024-11-08

**Authors:** Cinzia Di Franco, Eleonora Macchia, Michele Catacchio, Mariapia Caputo, Cecilia Scandurra, Lucia Sarcina, Paolo Bollella, Angelo Tricase, Massimo Innocenti, Riccardo Funari, Matteo Piscitelli, Gaetano Scamarcio, Luisa Torsi

**Affiliations:** ^1^ Institituto di Fotonica e Nanotecnologia (IFN) , Consiglio Nazionale delle Ricerche (CNR) CNR IFN Bari 70126 Italy; ^2^ Dipartimento di Farmacia‐Scienze del Farmaco Università degli Studi di Bari “Aldo Moro” Bari 70125 Italy; ^3^ Centre for Colloid and Surface Science at Università degli Studi di Bari Aldo Moro Bari 20125 Italy; ^4^ Dipartimento di Chimica and Centre for Colloid and Surface Science Università degli Studi di Bari Aldo Moro Bari 20125 Italy; ^5^ Dipartimento di Chimica Università degli Studi di Firenze INSTM Consortium ℅ Dip. Chimica Via della Lastruccia 3–13 Sesto Fiorentino I‐50019 Florence Italy; ^6^ Dipartimento Interateneo di Fisica Università degli Studi di Bari Aldo Moro Bari 70125 Italy; ^7^ Istituto di Intelligenza Meccanica Scuola Superiore Sant'Anna, Via G. Moruzzi, 1 Pisa 56124 Italy; ^8^ CNR‐ Istituto Nanoscienze c/o Scuola Normale Superiore Pisa 56127 Italy

**Keywords:** antibody‐capturing‐layers, anti‐IgM, electric‐field‐cycling, electrolyte‐gated‐organic field‐effect‐transistors(EGOFETs), Kelvin‐Probe‐Force‐Microscopy (KPFM), protein‐physisorption, Single‐Molecule‐With‐a‐Large‐Transistor(SiMoT), zeta‐potentials

## Abstract

The electric dipoles of proteins in a biolayer determine their dielectric properties through the polarization density P. Hence, its reproducibility is crucial for applications, particularly in bioelectronics. Biolayers encompassing capturing antibodies covalently bound at a biosensing interface are generally preferred for their assumed higher stability. However, surface physisorption is shown to offer advantages like easily scalable fabrication processes and high stability. The present study investigates the effects of electric‐field (EF)‐cycling of anti‐Immunoglobulin M (anti‐IgM) biolayers physisorbed on Au. The impact of EF‐cycling on the dielectric, optical, and mechanical properties of anti‐IgM biolayer is investigated. A reduction of the dispersion (standard deviation over a set of 31 samples) of the measured P values is observed, while the set median stays almost constant. Hence, physisorption combined with EF cycling, results in a biolayer with highly reproducible bioelectronic properties. Additionally, the study provides important insights into the mechanisms of dielectric rearrangement of dipole moments in capturing biolayers after EF‐cycling. Notably, EF‐cycling acts as an annealing process, driving the proteins in the biolayer into a statistically more probable and stable conformational state. Understanding these phenomena enhances the knowledge of the properties of physisorbed biolayers and can inform design strategies for bioelectronic devices.

## Graphical abstract

The effects of electric field cycling (EF‐cycling) on anti‐IgM layers physisorbed on Au, are studied. Physisorption of anti‐IgM lowers the Au work function, while a reduced dispersion of the sample's overall dipole moment, occurs after cycling, indicating that the electric field serves as an annealing process, guiding the physisorbed proteins toward a more stable state.

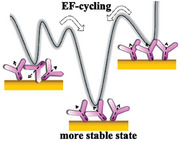

## 1 Introduction

The investigation of the dielectric properties of proteins, particularly as a deposited biolayer, holds significant importance due to their implications in diverse domains including bioelectronic sensing applications. Antibodies frequently possess a non‐zero electric dipole moment, µ, which makes them responsive to an external electric field (EF).^[^ ^1^ ^]^ When the proteins are deposited on a substrate to form a biolayer, this film encompasses a spatial distribution of dipoles associated with each deposited antibody. The resultant of these dipoles, i.e., the overall net dipole moment per unit volume or polarization P, characterizes the dielectric, or more in general the bioelectronic, properties of the film. These properties can be measured, for instance, by assessing the biolayer surface potential energy, *Φ*_S_, by Kelvin Probe Force Microscopy (KPFM).^[^ ^2^ ^]^ In fact, |P| corresponds to the surface charge σ_S_ which, in the simple case of a planar surface, is proportional to *Φ*_S_, through $P=\frac{\varepsilon }{e\;d}\Phi_{S}$ with *ε* being the dielectric constant of the medium, *e* the elemental charge, and *d* the effective thickness of the dielectric layer.

Understanding how such biolayers interact with an external EF can unravel insights into their dielectric properties defining their stability, conformational changes, and interactions. Despite the high relevance of these aspects, very few studies are available, and a complete understanding remains elusive. Furthermore, this knowledge is integral for designing bioelectronic devices where antibodies capturing layers serve as sensing elements, facilitating advancements in medical diagnostics and bioelectronics in general. Despite ongoing research efforts, the absence of a general model underscores the complexity of protein interactions with an external electric field, emphasizing the need for further exploration and refinement in this area.

Another relevant aspect is the biolayer deposition approach. While several methods for bio‐functionalizing a detecting interface have been proposed, physisorption is among the most convenient in all respects. However, there is an ingrained belief that a sufficiently stable capturing layer needs to be formed by antibodies covalently bound to a substrate. Another strong conviction is that a fully ordered layer is needed to enable high sensitivity. Both these aspects have driven the design of biosensing interfaces, dominated by covalently and orderly arranged antibodies in a capturing layer.^[^ ^3^, ^4^, ^5^ ^]^ Despite these convincements, antibodies adhering to solid surfaces through physisorption have been proven to retain full functionality, offering unique properties for applications in bioelectronics.^[^ ^6^, ^7^ ^]^ Moreover, it has been demonstrated that very high‐performance immunometric biosensing can be reached also with physisorbed capturing biolayer.^[^ ^8^, ^9^ ^]^

It is received that the irreversible protein physisorption leads to the formation of a monolayer due to repulsive interactions between the already physisorbed molecules and those in solution at physiological ionic strength, *i*_s_.^[^ ^10^ ^]^ These layers typically have a height of <10 nm, predominantly adopting a nearly end‐on configuration.^[^ ^8^ ^]^ At high ionic strength, weak, long‐range attractive interactions dominate^[^ ^11^ ^]^ while the balance between long‐range attraction and short‐range repulsion stabilizes the biolayer.

Although several studies deal with the investigation of how to orient proteins through an electric field interacting with their dipole moment,^[^ ^1^, ^12^, ^13^ ^]^ the use of electric fields acting like a thermal annealing process to stabilize the system, as demonstrated for instance in the assembling dispersions of colloids and nanoparticles, is interesting.^[^ ^14^ ^]^ An annealing typically involves heating a material to accelerate internal diffusion rates and grain growth while minimizing defects and strains. Such a procedure drives the material microstructure toward equilibrium, a desirable outcome in many cases. Annealing finds extensive use across many fields from inorganic materials to macromolecules. While accelerated annealing is often achieved by injecting energy into the system through external fields, an alternative approach to induce out‐of‐equilibrium fluctuations involves cycling the electric field (EF‐cycling). This method bears conceptual resemblance to temperature cycling utilized in the heat treatment annealing of several materials, mostly inorganic. A notable example of this approach is provided by the colloidal crystal monolayers annealing with an EF‐cycling.^[^ ^15^ ^]^ It is important to note that EF cycling cannot be considered a means to align the protein's dipole moments with the EF, as the electric‐filed phase changes at a regular pace. Hence EF‐cycling cannot lead to a more ordered protein layer by simply interacting with their dipole moments. It is also relevant to point out that among antibodies, anti‐immunoglobulins such as anti‐IgM are particularly important as they play a crucial role in many infection detections and disease diagnostics. They also often serve as model systems for designing properly functioning bioelectronic sensors.

In this paper, we undertake a comprehensive investigation of the dielectric, optical (infrared), and mechanical properties of an anti‐IgM layer physisorbed on gold before and after a treatment involving exposure to EF‐cycling. It is worth anticipating that, to avoid degradation of the biolayer, the voltage range spanned during the EF‐cycling is restrained to a region in which no faradaic electrochemical current is measured. The potentiometric characterization is carried out via three different techniques, namely: KPFM,^[^ ^11^ ^]^ Electrolyte‐Gated Organic Field‐Effect Transistors (EGOFETs),^[^ ^16^ ^]^ and zeta (ζ)‐potential. Additionally, the absence of alterations in the protein's secondary structure as well as their nanomechanical properties induced by EF‐cycling is assessed by Polarization Modulation Infrared Reflection‐Absorption Spectroscopy (PM‐IRRAS),^[^ ^17^ ^]^ force‐distance‐spectroscopy (F–D),^[^ ^18^ ^]^ and Atomic Force Microscopy (AFM), respectively.

The present study proves that the main effect of EF‐cycling on a physisorbed anti‐IgM layer is to reduce the dispersion (taken as one standard deviation) associated with P (proportional to *Φ*_S_/e), as well as with the threshold voltage *V*_T_ measured with the EGOFET and the ζ‐potential. Notably, in all these cases, the median across the inspected sample ensemble remains almost constant.

## 2 Results and Discussion

### 2.1 Anti‐IgM Biolayers Potentiometric Changes Upon EF‐cycling

#### 2.1.1 Kelvin Probe Force Microscopy Shifts

KPFM assesses the surface potential, SP, which is equal to *Φ*_S_/*e* with *Φ*_S_ being the surface contribution to the work function energy *Φ* of the inspected material. Indeed, the presence of a physisorbed layer on Au is known to affect its surface potential energy *Φ*_S,Au_. As an instance, thiolate self‐assembled monolayers (SAMs) adsorbed onto a gold surface, shift its surface potential lowering or increasing the work function, depending on the local average dipole moment orientation.^[^ ^19^ ^]^ Differently from thiolate SAMs, a physisorbed layer of proteins is not characterized by ordered dipoles, nor is there a net preferential orientation induced by a chemisorbing moiety on Au. Nonetheless, the entire layer, encompassing a volume distribution of dipoles µ, is characterized by an overall net layer polarization P.

**Figure 1:**
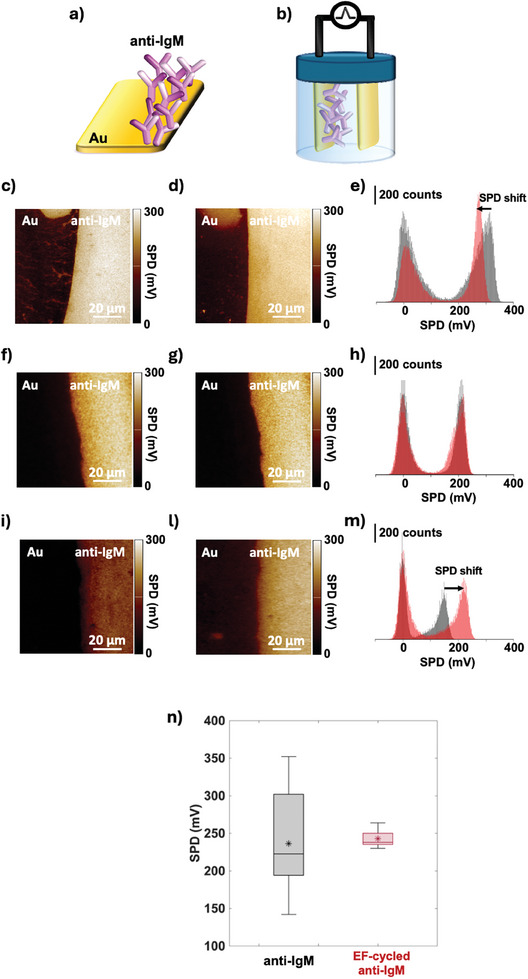
a) Schematic representation of an anti‐IgM layer physisorbed on the Au substrate evidencing the sharp Au/anti‐IgM interface. b) Representation of the cell, filled with deionized water, where the cycling of the electric field is carried out by sweeping the potential, applied between the anti‐IgM functionalized electrode and a gold counter electrode, in the range [0.1 V, −0.5 V] for 20 cycles. c) KPFM image of a pristine anti‐IgM layer at the interface with the bare gold substrate. d) KPFM image of the sample area inspected in panel c) after the EF‐cycling. e) Comparison of the SPD histograms of images b) and a), showing the shift to larger values of the SPD distribution after the EF‐cycling for this sample. The histogram of the pristine sample is black while that after EF‐cycling is red. f–h): same as c), d), e) showing that the SPD distribution remains approximately constant after the EF‐cycling for this sample. i,l,m): same as (c–e) showing the shift to smaller values of the SPD distribution after the EF‐cycling for this sample. n) The boxplot of the SPD measured on 10 different Au/anti‐IgM samples before (black‐symbol) and after (red‐symbol) EF‐cycling. The median value of the set is given as a continuous line in the light‐gray (pristine sample) or light‐red (after EF‐cycling) shaded box, while the average value of the set is marked with a star symbol.

**Figure** **1** shows representative KPFM images along with the relevant Surface Potential Difference (SPD) distribution histograms, before and after EF‐cycling. The inspected portion of the sample always encompasses the sharp interface (schematically featured in Figure 1a) between the gold substrate and a region coated with the physisorbed anti‐IgM (see Experimental Section for details). Such an interface is seen as a sharp change in SP. The latter is particularly relevant because gold is taken as the surface potential signal reference in KPFM measurements so SPD is the difference between the two SP values measured across the Au/anti‐IgM interface. The SPD parameter has been shown to be a very robust parameter that results in highly reproducible and reliable KPFM measurements.^[^ ^7^, ^20^ ^]^ Specifically, in these studies, a very detailed analysis is provided, showing that a KPFM investigation of anti‐immunoglobulins physisorbed biolayers exhibits an SP signal drifting with long exposure times to ambient atmosphere. This is ascribed to adventitious contamination. However, the exact same trend is also measured on the Au portion of the sample serving as a reference. Hence the SPD is invariant and proven to be a very robust parameter. To further support this evidence already published but on different systems, the KPFM data for an Au/anti‐IgM sample exposed to ambient atmosphere for several hours and subjected to several washings is provided in Figure S1 and described in Section S1 of the Supporting Information file. These control experiments, carefully executed according to the protocol detailed in the Experimental Section, clearly show no appreciable shift in the SPD values measured over 24 h of continuous exposure of the sample to the ambient atmosphere. Meanwhile, the sample was subjected also to five subsequent washing steps in deionized water. These also do not shift the SPD values. Such compelling evidence rules out the contribution of any spurious effects in the measured KPFM SPD shifts and definitively confirms the reliability of the KPFM measurements performed in our laboratories.

Figure 1b shows the setup where the EF‐cycling of the sample featured in Figure 1a, is carried out. Here, both the Au counter electrode and the Au/anti‐IgM samples are immersed, in deionized water (pH ≈ 5.5, *i*_s_ ≈ 5 µm), facing each other at a fixed spacing. The cell is connected to a potentiostat which applies a sweeping potential in the range [0.1 V, −0.5 V] at a scan rate of 100 mV s^−1^, for 20 cycles. To control the stability of the sample, the inspected voltage range is selected within the electrochemical inert region to prevent any electrochemical reaction in which a faradaic current flow at the electrode is associated. These processes are in fact known to degrade the sample. The negligible contribution of the faradaic current during the EF‐cycling is assessed by measuring the gate leakage current flowing in the EGOFET device (vide infra), Since no faradaic process is to be controlled the use of a reference electrode (e.g., Ag/AgCl) during the cycling, is unnecessary.^[^ ^21^ ^]^

Figure 1c shows the KPFM image of the Au and the Au/anti‐IgM regions and an average SPD = 306 ± 27 mV is measured. This is the baseline signal and it is associated with a lowering of the surface potential energy, *Φ*_S,biolayer_, and of the work function, *Φ*_biolayer_, occurring when the anti‐IgM layer is deposited on the gold. Afterward, the same Au/anti‐IgM sample undergoes an EF‐cycling, and the sample is re‐inspected, targeting under the exact experimental conditions, the same portion imaged before the EF‐cycling. These data are shown in Figure 1d. An average SPD of 252 ± 9 mV can be measured after EF‐cycling which, in this specific sample, induces a shift in the Au/anti‐IgM surface potential voltage, *Φ*_S,biolayer_/e, by – (54 ± 28) mV, hence a reduction of SPD is measured. The SPD distribution histograms for the pristine (black) and the EF‐cycled (red) samples are shown in Figure 1e.

While for the sample featured in Figure 1c–e, *Φ*_S,biolayer_ decreases after EF‐cycling, there are also samples where the *Φ*_S,biolayer_ stays constant or increases. In Figure 1f–h the KPFM images which feature a sample where SPD stays constant, are shown, while in Figure 1i–m a sample where SPD increases, is presented. The SPD average and median values measured on the whole set of 10 samples are given in Figure 1n as black (pristine) and red (cycled) boxplots. Before cycling, the black boxplot shows an average of 236 mV and a very large standard deviation of 70 mV. After cycling the Au/anti‐IgM surface potential voltage falls at an average of 243 ± 11 mV. As it is apparent, the standard deviation is significantly smaller after EF‐cycling, hence the error, relative change, Δ*ε*/*ε* = [(*ε*_after‐cycling_‐ *ε*_before‐cycling_) / *ε*_before‐cycling_] = −84% sees a large reduction. On the other hand, the average *Φ*_S,biolayer_ value is invariant within the error bars that are larger before cycling. This means that after EF‐cycling, the *Φ*_S,biolayer_/e, or equivalently P, converge to a set of much less spread values and hence, the reproducibility of P is greatly improved. As a final note, it is important to clarify that while the *Φ*_S,biolayer_/e values upon EF‐cycling can either decrease or increase compared to Au, depending on the sample, *Φ*_S,biolayer_/e always decreases (compared to Au) when the biolayer is deposited on the gold substrate. This specific feature will be addressed later in the text (see Section 2.5).

#### 2.1.2 The EGOFET Threshold Voltage (*V*_T_) Shift upon EF‐cycling

While in potentiometric KPFM measurements, a SPD shift is seen upon physisorption of a monolayer on gold or upon EF‐cycling, in the case of an organic field‐effect transistor (OFET), a shift in the threshold voltage (*V*_T_) is observed when the work function of the gate is changed.^[^ ^22^ ^]^ Relevantly, SPD and *V*_T_ are comparable figures, being both connected to *Φ*/e as will be detailed in Section 2.5.

**Figure 2:**
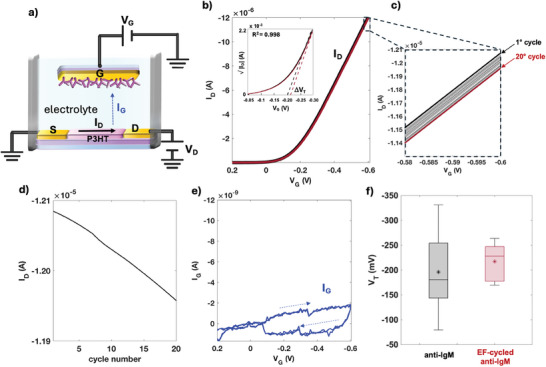
a) Schematics of an electrolyte‐gated organic field effect transistor (EGOFET). A gate electrode (G) (0.25 cm^2^) covered with the physisorbed anti‐IgM layer is capacitively coupled to a P3HT FET channel contacted through the source (S) and drain (D) interdigitated pads. b) *I*_D_ versus *V*_G_ transfer characteristics (*V*_G_ swept from 0.2 V to −0.6 V while *V*_D_ is kept constant at −0.4V) are measured by modulating the *I*_D_ current with the anti‐IgM functionalized gate. The first trace (measured back and forth) is evidenced with a thick black line. The transfer characteristics are measured for 20 consecutive times (cycling) and the 20th curve is shown as a thick red curve, while all the traces recorded in between are shown as thin black lines. The *V*_T_ threshold voltage extracted from one of the transfer characteristics is also shown in the inset. c) Magnification of the currents measured in the high gate‐voltage region, where the transconductance of the device is maximized. d) Transient evolution of the *I*_D_ point‐value current measured at *V*_G_ = −0.6V during the cycling protocol. e) Measurement of the 20‐gate‐leakage current curves, *I*_G_ versus *V*_G_, measured during the EF‐cycling to show that a negligible Faradaic current flows in the direction perpendicular to the gate. f) Boxplots represent the distributions of the *V*_T_ shifts upon the EF‐cycling from ten replicates. The black symbols are relevant to the transfer characteristics measured during the first cycle, while the red ones are relevant to the last curves acquired. The median value is given as a continuous line in the light‐gray or light‐red shaded box, while the average value is marked with a star symbol.

EGOFETs,^[^ ^16^ ^]^ that are OFET operated in an electrolyte, have been widely used to study biological systems and the effect of affinity binding events, by measuring the associated transistor *V*_T_ shift.^[^ ^23^, ^24^, ^25^, ^26^, ^27^, ^28^, ^29^, ^30^ ^]^ Recently, changes in the *V*_T_ of an Au/anti‐IgM gate electrode have been also correlated with shifts in the electrode SPD.^[^ ^7^, ^20^ ^]^ An EGOFET is a three‐terminal device consisting of source (S) and drain (D) electrodes connected via an organic semiconductor channel, facing the gate (G) electrode, biofunctionalized with a physisorbed layer of anti‐IgM. A schematic of an EGOFET structure is depicted in **Figure** **2**a. To ensure a fair comparison with the KPFM data, the Au‐gate electrode is biofunctionalized following exactly the same protocol. The only difference is that the EGOFET entire gate surface (and not just half of it) is covered by the physisorbed anti‐IgM layer. Patterning the sample into two regions is unnecessary for EGOFETs because the grounded source electrode serves as the reference for the potentiostatic measurement, given that the p‐type EGOFET device is operated in the common source measuring configuration. An exact comparison between the two measuring systems is provided in Section 2.5.

Both the channel and the gate are immersed in an aqueous electrolyte (deionized water HPLC‐grade, pH ≈ 5.5, *i*_s_ ≈ 5 µm). Upon biasing, a charge‐double‐layer (CDL) with a capacity as high as tens of microfarad per square centimeter, is formed both at the gate/electrolyte and at the electrolyte/organic semiconductor interfaces. The channel conductivity is modulated by capacitive coupling with the gate electrode, actuated via the electrolyte. The very low i_s_ maximizes the Debye length, ensuring a largely unscreened biological layer, thereby enhancing the response of the device to changes occurring within the biolayer. The transistor current‐voltage (*I*_D_ vs *V*_G_) transfer characteristic curves are recorded at a constant *V*_D_ of −0.4 V, by sweeping *V*_G_ according to the EF‐cycling protocol as detailed in the Experimental Section. In these experiments, the EF‐cycling involves the iterative measurement of the 20 consecutive transfer curves shown in Figure 2b. In the inset, *V*_T_ is graphically extracted from the $\sqrt{I_{D}}$ versus *V*_G_ plot of the first (black) and last (red) cycle to measure the *V*_T_ shift before and after completing the EF cycling.^[^ ^4^ ^]^ In Figure 2c a zoom into the higher current region is offered to better display how the current changes when going from the 1st to the 20th cycle. The initial current level, depicted as a thick black curve, is compared to the transfer characteristic recorded after 20 cycles (red curve). In between, all the other measured curves are shown, and *V*_T_ is extracted from each one of these 20 curves. In Figure 2d, the point values of the I_DS_ current at *V*_G_ = −0.6 V, show a significant current decrease along with a shift of the transfer characteristics toward a more negative *V*_G_. The overall shift upon EF‐cycling in this specific sample is – 20 mV.

In Figure 2e the gate leakage currents *I*_G_ in the *V*_G_ range of the EF‐cycling are shown. Those 20 curves are measured during the EF‐cycling, and they show how a negligible faradaic *I*_G_ current, more than three orders of magnitude lower than the *I*_D_ FET‐channel current (Figure 2b), flows in the device. The shape of the *I*_G_ curves is typical of a capacitive current that arises from the charging and discharging of the charge double layer at the electrode/electrolyte interface. So, no sign of Faradaic current is seen, which has been demonstrated to be very important for the stability of the device.^[^ ^31^ ^]^ The occurrence of a very low faradaic current, also further confirms the unnecessary use of a reference electrode in the potentiometric measurements.

In Figure 2f the *V*_T_ dispersion over 10 different EGOFETs with an Au/anti‐IgM gate, are shown as boxplots before (black symbol) and after EF‐cycling (red symbol). The *V*_T_ average value registered on anti‐IgM samples before cycling is – (200 ± 80) mV shifting to – (220 ± 40), afterward. In agreement with the KPFM investigation, the standard deviation *ε* is reduced from 80 mV to 40 mV, with a Δ*ε*/*ε* = _–_ 50%. It is worth mentioning that the shift of *V*_T_ with an Au gate over the same EF‐cycling is proven to be only within 5%.^[^ ^31^, ^32^ ^]^ Relevantly, while a bare‐gold gate has a *V*_T_ of – (135 ± 8) mV, with the anti‐IgM coating it goes to – 200 ± 80 mV, with a shift of – (65 ± 80.4) mV. The shift toward more negative *V*_T_ (with a p‐type semiconductor EGOFET) is seen when the gate *Φ* becomes lower.^[^ ^33^ ^]^ Hence also the EGOFET data proves, in agreement with KPFM, that the physisorption of the anti‐IgM layer lowers the electrode *Φ*. On the other hand, the EF‐cycling of the EGOFETs can, alike the KPFM samples, either lower or increase *V*_T_, depending on the sample.

#### 2.1.3 ζ‐Potential Assessment upon EF‐cycling

**Figure 3:**
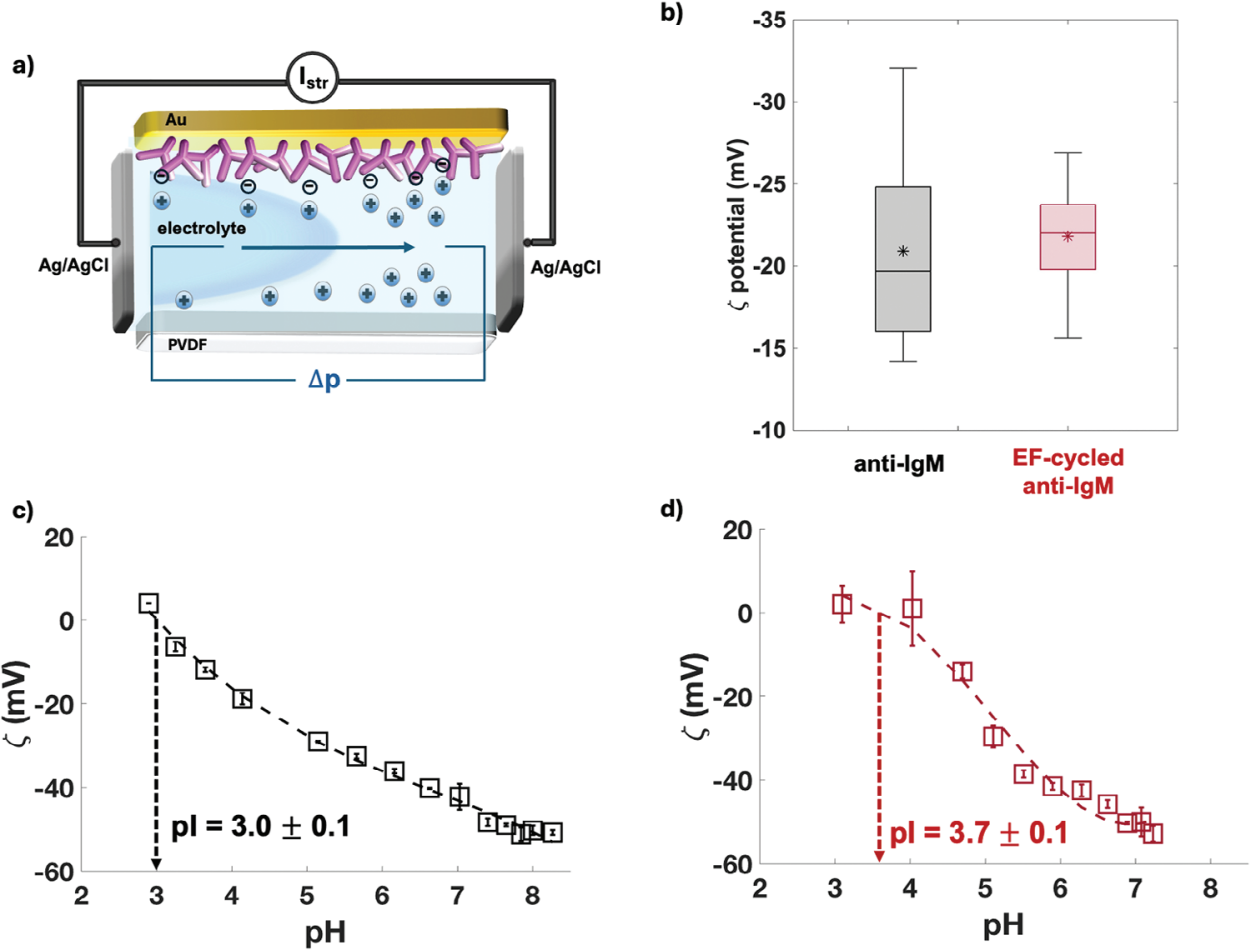
a) Scheme of the SurPASS 3 adjustable gap cell used for the ζ‐potential measurements. b) Boxplots showing the average ζ−potential shifts over 11 replicates, assessed before (black symbols) and after (red symbols) the EF‐cycling. Extracted ζ‐potential versus pH of the pristine c) and cycled d) anti‐IgM sample; on each sample, data are taken in duplicates, and error bars are one standard deviation.

The third potentiometric technique involves the ζ‐potential assessment of the Au‐/anti‐IgM surface. This is carried out to measure the surface charge present on the sample at a given pH, providing insights into the physisorbed protein dielectric configuration. **Figure** **3**a features the schematics of the cell used for these measurements. The ζ‐potential is measured by recording the streaming current in a KCl electrolyte (*i*_s_ = 10 mm and pH = 5.5) flowing between two Ag/AgCl electrodes connected at the cell inlet and outlet.^[^ ^34^, ^35^, ^36^ ^]^ The electrolyte flow is generated by the pressurization of the liquid reservoir with atmospheric air that causes electrical charge separation in the flow direction as the electrolyte streams through the measuring cell. The resulting streaming current is measured by the two Ag/AgCl electrodes. During a measurement, the pressure difference across the measuring cell (Δ*p*) and the streaming current (*I*_str_) are recorded through a pressure control unit and an electronic circuit with low internal resistance, respectively.

The measured values of Δ*p* and *I*_str_ serve to calculate the ζ‐potential according to Equation 1:

(1) $$\zeta =\frac{dI_{str}}{d\Delta p}\cdot \frac{\eta }{\varepsilon }\cdot \frac{L}{A}$$

with *η* being the viscosity, *ε* the dielectric constant L and A the length, and the cross‐section of the streaming channel, respectively.

The ζ‐potential of 11 pristine Au/anti‐IgM samples is measured returning an average value at pH 5.5 and *i*_s_ = 10 mm of – (21 ± 6) mV while after cycling the average is equal to – (22 ± 3) mV as it is shown in Figure 3b. The average value is again invariant while the Δ*ε*/*ε* reduction is – 50%, the same figure measured for EGOFETs. Also in this case, depending on the sample, the ζ‐potential can increase or decrease, after the EF‐cycling.

The isoelectric point (pI) of a specific Au‐anti‐IgM layer is measured with a pH titration carried out by adjusting the electrolyte pH with 50 mm HCl and 50 mm NaOH solutions. The pI of a specific pristine anti‐IgM sample is 3.0 ± 0.1 (Figure 3c), and it becomes 3.7 ± 0.1 after cycling (Figure 3d). The error bars of the two points at lower pH are, apparently, the largest. Since those data are the most relevant for assessing the zero ζ‐potential the average uncertainty of the first two points is used in the extrapolation of the pI values, which results in an error of 0.1. The pI values extracted indicate that at pH 5.5 the pristine Au/anti‐IgM surface is overall negatively charged and after cycling it becomes less negative. For this specific sample, upon EF‐cycling a shift toward more basic pH values along with a ζ‐potential better saturation at low pH values, is seen. This evidence can be related to a different acidic strength of the Au/anti‐IgM surface inspected: before cycling, the sample acts as a stronger acid with a complete deprotonation at a lower pH value.

### 2.2 Thickness Estimate of Anti‐IgM Biolayers

Anti‐IgM biolayers are also investigated by a surface plasmon resonance (SPR) and the experiments are shown in Figure S2a (Supporting Information). These data are compatible with a physisorbed dense and compact single layer of immunoglobulins,^[^ ^8^ ^]^ proving that, like anti‐IgG, 10^11^–10^12^ proteins are physisorbed on 1 cm^2^ area, resulting in a density of 10^4^ proteins µm^−2^.^[^ ^6^, ^8^, ^37^ ^]^ The density does not change even after washing the sample in H_2_O. Moreover, a resulting average anti‐IgM biolayer thickness of 9 ± 2 nm is estimated from the modeling of the SPR data (Figure S2b, Supporting Information). AFM is also used to estimate the film thickness resulting in an average of 6.7 ± 1.4 nm (Figure S3, Supporting Information), and, notably, the two independently measured values, are comparable within the errors. The anti‐IgM film thickness measured averaging both approaches is ≈ 8 nm, and the uniformity of the deposit (proven by AFM inspection on different areas of the biolayer) complies with a physisorbed biolayer composed of a single layer of antibodies laying nearly edge‐on to the Au substrate.

### 2.3 Anti‐IgM Biolayers PM‐IRRAS Characterization

A further characterization of the Au/anti‐IgM layer involves a PM‐IRRAS study. The peptide chains, which are the backbones of proteins, give rise to characteristic features in the mid‐infrared spectrum.

**Figure 4:**
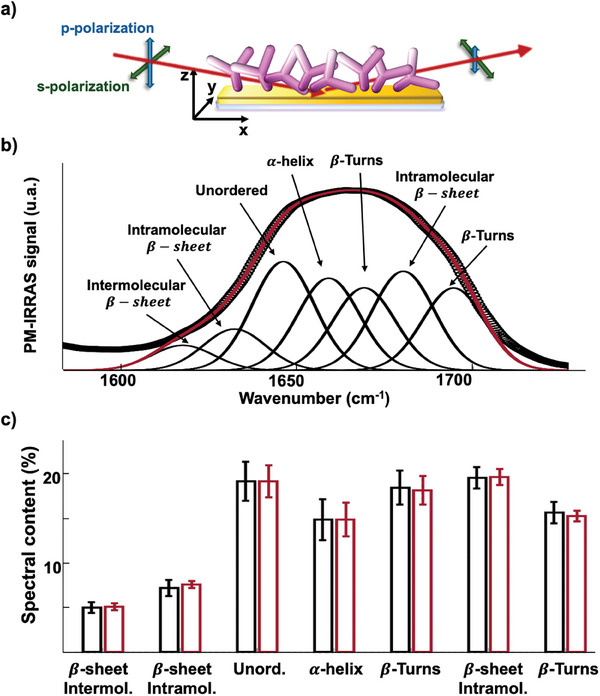
a) Schematics of the PM‐IRRAS apparatus showing the anti‐IgM sample physisorbed on an Au‐plated reflective substrate. The red arrow represents the IR radiation reflected under grazing angle incidence at 82° by the sample surface. The green and blue arrows represent s‐ and p‐polarizations, respectively. The shortening of the blue arrow after reflection indicates the selective absorption of p‐polarized IR light as predicted by selection rules. b) Representative PM‐IRRAS spectrum of anti‐IgM physisorbed on Au in the Amide I (≈1650 cm^−1^) spectral range. The experimental points (black markers) are perfectly reproduced by a fitting curve (red line) including seven Gaussian bands, representing the contribution to the protein secondary structure, from C═O stretching vibrations, as described in the text. c) Spectral content of Amide I contributions, expressed as a percentage of the area under each of the seven Gaussian peaks with respect to the total Amide I area. The black symbols are the data from as‐deposited samples, while the red ones are taken after the cycling. The values and error bars are computed as the average and one standard deviation over five samples.

The main bands are Amide I (≈1650 cm^−1^), due to the C═O stretching vibrations; Amide II (≈1550 cm^−1^) resulting from the N─H bending and C─N stretching vibrations; Amide A (≈3300 cm^−1^), which originated from the stretching vibrations of hydrogen‐bonded NH groups.^[^ ^38^ ^]^ Here we focus on the analysis of the Amide I band, which provides valuable information on the protein secondary structure. **Figure** **4**a shows the IR beam‐sample geometrical arrangement. A representative PM‐IRRAS spectrum of the physisorbed anti‐IgM samples, in the Amide I spectral range, is shown in Figure 4b. As usual, the second derivative spectra (not shown) were used to identify the main secondary structure contributions under Amide I.^[^ ^39^ ^]^ Accordingly, fitting all experimental spectra with seven Gaussian functions returns excellent reproduction of the Amide I band. We found the following contributions: intermolecular β‐sheet (1619 ± 1 cm^−1^), intramolecular β‐sheet (1633 ± 1 cm^−1^ and 1681 ± 1 cm^−1^), unordered (1646 ± 1 cm^−1^), α‐helix (1658 ± 1 cm^−1^), β‐turns (1669 ± 1 cm^−1^ and 1696 ± 1 cm^−1^). While no study on PM‐IRRAS of physisorbed anti‐IgM has been reported, our spectral assignments are consistent with similar analyses carried out on the infrared spectra of IgG‐like proteins.^[^ ^40^, ^41^, ^42^ ^]^

To study the effect of EF‐cycling on the secondary protein structure, we compare in Figure 4c the histograms of the PM‐IRRAS spectral contents associated with each Amide I component of pristine and cycled samples. Significantly, no substantial difference is observed, suggesting that the secondary structure of anti‐IgM proteins highly packed in the Au/anti‐IgM biolayer is almost insensitive to the application of a cyclic external electric field. Also, no relevant change is observed in the other spectral regions.

### 2.4 Anti‐IgM Biolayers Nanomechanical Characterization upon EF‐cycling

**Figure 5:**
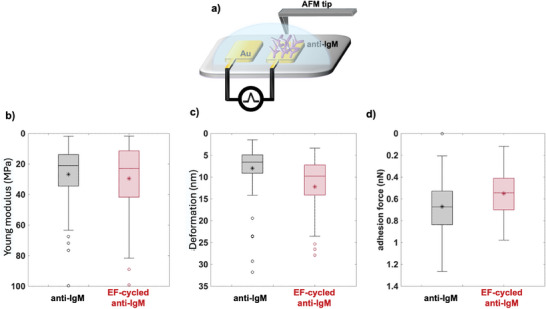
a) Schematic illustration of the custom AFM liquid cell used for the force‐distance spectroscopy. Force‐distance curves are collected in 4 × 4 arrays on different areas of an Au/anti‐IgM sample in quintuplicate to characterize the protein layer before and after cycling. The boxplots show the distributions of the antibody layer mechanical properties before (black symbols) and after (red symbols) the cycling, namely: in panel b) the extracted Young's modulus, in c) deformation, and in d) the adhesion force. The median value is given as a continuous line in the light‐gray or light‐red shaded box, while the average value is marked with a star symbol.

Besides the potentiometric data and the topographic information gathered with the AFM scan, we have also investigated the effect of EF‐cycling on antibodies in the Au/anti‐IgM layer nano‐mechanic properties. This characterization is carried out in a liquid environment by (F–D)‐spectroscopy. The F–D analysis is performed in contact mode, using a customized liquid cell. Two gold pads (0.5 cm^2^), serving as counter and working electrodes are fabricated in proximity (≈ 2 mm apart) through a shadow mask, on a Si/SiO_2_ substrate. The working electrode surface is biofunctionalized with the physisorbed anti‐IgM layer. **Figure** **5**a shows the in situ liquid cell used for the EF‐cycling, which works similarly to the ex situ one featured in Figure 1e. The same EF‐cycling duty cycle is used as well. Specifically, the cell, designed for the in situ EF‐cycling inside the AFM microscope environment, features a design where the Au/anti‐IgM working electrode is easily available for the AFM scan. The cell is connected to a bi‐potentiostat which applies the EF‐cycling protocol used so far.

Force‐distance curves are recorded in contact mode over different areas of biofunctionalized samples in quintuplicate. The Johnson–Kendall–Roberts model^[^ ^43^ ^]^ is applied to the approach and retraction curves.^[^ ^44^ ^]^ Young's modulus, *E* (MPa), deformation, *D* (nm), adhesion force, and F (nN) are extracted from the curves collected before and after the protein cycling. An average Young's modulus of *E* = 27 ± 19 MPa is found for the as‐deposited anti‐IgM samples, in good agreement with the reported literature values for IgG‐like proteins.^[^ ^45^, ^46^, ^47^ ^]^ The data are shown as a black boxplot in Figure 5b. The cycled Au/anti‐IgM average is *E* = 30 ± 20 MPa (red boxplot), suggesting that the elastic modulus does not change upon EF‐cycling. Similarly, deformation (Figure 5c) and adhesion force (Figure 5d) mean values are comparable (*D* = 8 ± 5 nm, *F* = 0.7 ± 0.2 nN, respectively), to the corresponding values measured after cycling (*D* = 12 ± 9 nm, *F* = 0.6 ± 0.2 nN, respectively). Relevantly, no reduction of the data dispersion is measured as well.

### 2.5 Unified Description of the *Φ*_S_, *V*_T,_ and ζ‐potential Potentiometric Data

It is clear at this stage that the only experiments that proved a sizable change in a property (*Φ*_S_, *V*_T_, or ζ‐potential) over the assessment of 31 samples upon EF‐cycling, are the potentiometric ones. In fact, none of the *Φ*_S_, *V*_T_, or ζ‐potential shifted their average value, but all showed a very important reduction of the dispersion of the data measured as one standard deviation. However, the three different potentiometric approaches, KPFM, EGOFET, and ζ‐potential, address slightly different aspects of the dielectric properties of the Au/anti‐IgM biolayer and while the conditions in the two sets of KPFM and EGOFET experiments were kept as similar as possible, some differences were inevitable. It is very important at this stage to assess if, despite the differences, the potentials measured can be described in a unified framework. To this end, all the average measured potentials (*Φ*_S_, *V*_T_, or ζ‐potential), and their dispersions over 10 or more samples for each technique, are gathered in **Table** **1**.

**Table 1:** Summary of the average potentiometric data along with errors or dispersions (one standard deviation) for the Au and Au/anti‐IgM samples measured by KPFM, EGOFET, and ζ‐potential.

	KPFM SPD = *Φ*_S_/*e* (dried samples) 10 samples	EGOFET *V*_T_ (pH ≈ 5.5, *i*_s_ ≈ 5 µm) 10 samples	ζ‐potential (pH = 5.5, *i*_s_ = 10 mm) 11 samples	Average error over the whole set of 31 samples
Shift upon anti‐IgM deposition on Au	–	−(65 ± 41) mV	–	–
Au/anti‐IgM before‐cycling (*ε*_R_)^a)^	236 ± 70 mV (30%)	−(200 ± 80) mV (40%)	−(21 ± 6) mV (28%)	33%
Au/anti‐IgM after‐cycling (*ε*_R_)^a)^	243 ± 11 mV (4%)	−(220 ± 40) mV (18%)	−(22 ± 3) mV (14%)	12%
Δ*ε*/*ε* ^b)^	−84%	−50%	−50%	−61%

^a)^ relative error

^b)^ Δ*ε*/*ε* = [(*ε*_after‐cycling_‐ *ε*_before‐cycling_) / *ε*_before‐cycling_]

Table 1 provides, hence, a synoptic summary of all the potentiometric data measured so far, along with the dispersion errors, both the relative errors, *ε*_R_, and the Δ*ε*/*ε*.

**Figure 6:**
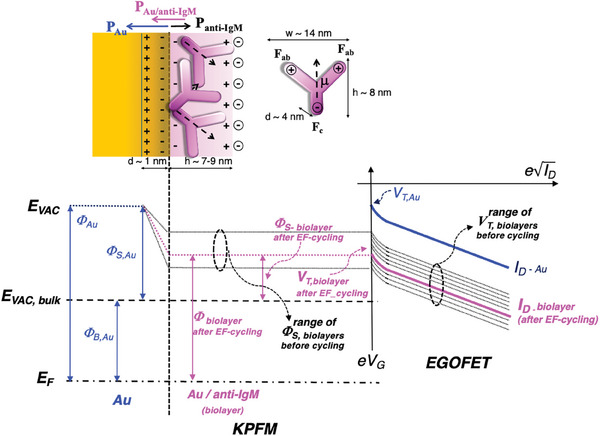
Schematic representation of the energy diagram levels of the Au/anti‐IgM samples. The work function (*Φ*), and surface potential energy (*Φ*_S_) are shown for the Au sample before and after the anti‐IgM physisorption. The bulk potential energy (*Φ*_B_) for Au is also shown along with the vacuum levels E_VAC_ and the Femi level *E*_F_. The corresponding energy levels for the Au/anti‐IgM biolayer before and after the EF‐cycling, are shown as well. The blue color code identifies the energy levels for the bare gold. The magenta lines show the energy levels after cycling while the black ones refer to the highly dispersed surface potential energy levels measured on different samples, before cycling. The corresponding threshold voltage shifts, *V*_T_ (given as energy levels, *e*⋅*V*_T_) measured with the EGOFET devices and extracted from the $\sqrt{I_{D}}$ versus *V*_G_ plots, are also shown on the rightmost part of the figure. On the top‐right, a schematic representation of a possible structure of an Au/anti‐IgM biolayer is shown along with a schematic structure of an antibody featuring both the antigen‐binding, *F*_ab_, and the constant, *F*_c_, fragments.

Before proceeding with the unified description, a schematic qualitative energy level diagram is given in **Figure** **6**, showing how the *Φ* and *Φ*_S_ energy values change upon physisorption of the anti‐IgM biolayer on the Au substrate and how they further change upon EF‐cycling.

In the top left of Figure 6 a schematic view of the Au/anti‐IgM biolayer energy level structure is shown. Additionally, on the left side, a representation of the main structure of an antibody is provided. This consists of two identical *F*_ab_ (fragment antigen‐binding) arms and one constant fragment, *F*_c_, portion, forming the typical “Y” shape with typical dimensions being ca. 14 nm × 8 nm × 4 nm (*d* ≈ 4 nm being the width of the antibody). The *F*_ab_ fragments contain variable regions that bind specifically to antigens. The isoelectric point, pI, of the *F*_ab_ fragment is generally higher than that of the *F*_c_ fragment and of the entire antibody. In a physiological buffer with a pH falling between the pI of the *F*_ab_ and *F*_c_ fragments, the *F*_ab_ fragment is positively charged while the *F*_c_ fragment is negatively charged. This configuration allows the antibody to have an associated electric dipole moment µ being a vector pointing from the negatively charged *F*_c_ to the positively charged *F*_ab_.^[^ ^1^ ^]^

In the bottom portion of Figure 6, a graphical representation of the energy levels for the work function, *Φ*, and the surface potential energies, *Φ*_S_ of the Au/anti‐IgM system, is also provided. On the left, these levels are illustrated for the Au layer in blue, with *Φ*_Au_ = *Φ*_S,Au +_ *Φ*_B,Au_, *Φ*_B,Au_ being the bulk potential energy. Moving toward the right, the energy levels at the interface between the gold substrate and the physisorbed anti‐IgM layer are depicted. The KPFM data in Figure 1, prove that the biolayer physisorption lowers the *Φ*_S_ of Au. While the actual quantitative shift is sample‐dependent, in all cases, a Φ_S_ decrease is observed when the anti‐IgM layer is physisorbed on Au. This is featured by the downward bending of the vacuum levels, *E*_VAC_, delineated by two horizontal dotted black lines, ideally encompassing the different surface potential values (not shown) of each of the 10 as‐deposited (pristine) Au/anti‐IgM samples examined. The data presented in Figure 2, show (also on 10 different samples) that *V*_T_ shifts toward more negative values are measured when the anti‐IgM layer is added to Au. This shift is seen on p‐type EGOFETs when Φ_S_ decreases,^[^ ^33^ ^]^ and the corresponding *V*_T_ values are illustrated schematically in the rightmost part of Figure 6. Those associated with the *Φ* levels of the Au substrate are represented in blue, while those pertinent to the Au/anti‐IgM layer are depicted as black dotted lines. The *V*_T_ values are also schematically shown in Figure 6 as black dotted line curves, each one representing a different Au/anti‐IgM gate before EF‐cycling. The average values of the actual Φ_s_ and *V*_T_ potential measured over the 10 replicates for both the KPFM and the EGOFET systems are summarized in Table 1. Interestingly, these data are comparable within the error bars. A possible explanation for such an occurrence is provided in the following. The surface potential signal, SP = *Φ*_S_/e, measured by KPFM on the Au/anti‐IgM biolayer is given by:

(2) $$SP_{biolayer}\left(V\right)=\left(F_{tip}-F_{biolayer}\right)/e$$

with *Φ*_tip_ being the work function of the inspecting tip and *e* the elemental charge. The Au/anti‐IgM biolayer work function, *Φ*_biolayer_, extracted from the measured *Φ*_S_ signal and the known *Φ*_tip_, is:

(3) $$F_{\mathrm{biolayer}}\left(\mathrm{eV}\right)=F_{\mathrm{tip}}\left(\mathrm{eV}\right)-e\cdot SP_{\mathrm{biolayer}}\left(V\right)$$

On the patterned sample featuring the interface between the uncovered gold substrate and the anti‐IgM layer, the surface potential measured on the gold part, SP_Au_ (V) = (*Φ*_tip_ – *Φ*_Au_)/*e*, is taken as reference. Hence the relevant SPD value plotted in Figure 1, is given by:

(4) $$\mathrm{SPD}\;\left(V\right)=SP_{\mathrm{biolayer}}-SP_{\mathrm{Au}}=\left(\Phi_{\mathrm{Au}-}\Phi_{\mathrm{biolayer}}\right)/e$$

*V*_T_ in an EGOFET is the gate bias that needs to be applied to form a conductive channel in the FET; in unintentionally p‐doped organic semiconductors, such as P3HT, this occurs in the accumulation regime where *V*_G_ is more negative than flat‐band potential, *V*_FB_, given by:^[^ ^33^ ^]^

(5) $$V_{\mathrm{FB}}=\left(\Phi_{\mathrm{biolayer}}-\Phi_{P3\mathrm{HT}}\right)/e-Q_{\mathrm{is}}/C_{i}$$

with, *Q*_is_ being the interface charge density, and C_i_ the CDL capacitance (per unit area) at the interface between the gate and the electrolyte. If we take *Q*_is_ equal to zero, Equations 4 and 5 both correlate *Φ*_biolayer_ to a reference work function.

While the conditions in the two sets of KPFM and EGOFET experiments were kept as similar as possible, some differences were inevitable, namely: i) the KPFM SPD (V) data are measured using *Φ*_Au_ as a reference, whereas for the EGOFET, the reference is *Φ*_P3HT_; ii) the KPFM data are acquired from dried samples in air, while the EGOFET measurements are performed in deionized water.

As to the first point i) is concerned, for a solution‐processed P3HT a valence band edge, or equivalently a *Φ*_P3HT_, of 5.1 eV is reported^[^ ^48^ ^]^ which is comparable to *Φ*_Au_ as measured by photoemission spectroscopy on sputtered gold films.^[^ ^49^ ^]^ So both techniques measure the data with respect to an, incidentally, equivalent reference level.

To deal with the second point ii) the average surface charge density in the Au/anti‐IgM layer is estimated from the experimental SPD measured by KPFM to be:

(6) $$\mathrm{SP}D_{\mathrm{biolayer}}=d\cdot \sigma_{\mathrm{biolayer}}/\varepsilon$$

taking *d* = 7 – 9 nm as the average anti‐IgM thickness and *ε*, the Au/anti‐IgM layer dielectric constant, as ≈ 3.2, the resulting σ_biolayer_ is ≈ 2 effective electron charge in (10 nm)^2^, the latter being approximately the footprint of a single physisorbed anti‐IgM protein. Hence the effective charge density corresponds to approximately 2 electron charges per adsorbed anti‐IgM.

The ionic strength of the medium in contact with the inspected surface, which can condition its surface potential, is also considered. The length at which the electric field *Ψ* (*x*) in the CDL reduces by 1/e due to charge screening, namely the Debye length, λ_D_, is given by the equation:^[^ ^50^ ^]^

(7) $$\lambda_{D}=\sqrt{\varepsilon k_{B}T/2N_{A}e^{2}i_{s}}\;\mathrm{with}\;\;\;\psi \;\left(x\right)=\psi_{0}e^{-\frac{x}{\lambda_{D}}}$$

with *ε* being the dielectric constant of the solvent, *k*_B_ the Boltzmann's constant, *T* the temperature, *N*_A_ the Avogadro's number. In deionized water (*i*_s_ ≈ 5 µm, ε = 80) λ_D_ is equal to 138 nm, which is much longer than the actual anti‐IgM layer, estimated to be 7 – 8 nm high.^[^ ^8^ ^]^ Considering that the EGOFET is measured in deionized water, this implies that the charges at the Au/anti‐IgM surface are likely to remain largely unscreened in such an extremely low ionic strength environment. A very rough quantitative estimation can be given considering the volume of the anti‐IgM film (0.5 cm^2^ × 8 nm = 4 × 10^−7^ mL) at an i_s_ of 5 µm where ≈10^8^ ions can be found, which clearly leaves the Au/anti‐IgM layer deposited on a 0.5 cm^2^ electrode comprising overall 10^11^–10^12^ charges, not fully screened. As a side comment, this experimental setting is chosen to enable the detection of the binding effect, known to impact the dielectric properties of the capturing antibodies, in the condition where screening of the charges of a protein by the ions of the electrolyte solution is at a minimum.^[^ ^51^ ^]^ This is the reason why the *I*_D_ versus *V*_G_ transfer curves of the EGOFETs are measured in deionized water, while the binding occurs in the medium with a physiological i_s_.^[^ ^23^, ^29^, ^52^ ^]^

The KPFM data are obtained on an Au/anti‐IgM sample after thorough washing in deionized water, drying, and measurement in air, namely in a standard humid atmosphere. Here, a significant electrostatic unscreening of the surface is expected too, as the ionic conductivity of standard humid air can drop down to 10^−15^ S m^−1^,^[^ ^53^ ^]^ much lower than the 10^−6^ S m^−1^ of the deionized water used. Under the assumption that a great deal of unscreening is already reached in deionized water, it follows that this condition cannot become worse in the air. Consequently, the KPFM and EGOFET assessments of the Au/anti‐IgM surface, although conducted under different experimental conditions, can yield measured voltages that, within the margin of error, are similar. This is exactly what we measure as it can be better appreciated by comparing the KPFM and *V*_T_ potentials measured and listed in Table 1.

The measured ζ‐potentials, given also in Table 1, are, conversely, approximately one order of magnitude lower than those measured with the other two potentiometric techniques. We here investigate the origin of this difference that is connected to the necessarily different experimental conditions. While KPFM are measured in air and EGOFETs are assessed in deionized water, ζ‐potentials are necessarily measured in a much higher salinity buffer. As detailed also in the Experimental Section, the ζ‐potential is a measure of the electrokinetic potential at an interface. The measurement addresses the charges that are not stripped away from the inspected surface by the streaming current flowing through the cell during the measurement. Generally, the ζ‐potentials are connected to the structure of the Stern layer in a CDL.^[^ ^54^ ^]^ This layer, also known as the inner Helmholtz plane, refers to the rather compact layer of ions coming from the surrounding electrolyte solution, that are electrostatically attracted by a charged surface, in the case here under study, the negatively charged gold layer. The Stern layer is, hence, formed by tightly electrostatically bound ions, that are not stripped away by the flow of the streaming current. The ζ‐potential is, therefore, a measure of the electrical potential at the boundary between the Stern layer and the diffuse layer (outer Helmholtz plane) in a CDL, also addressed as the shared plane. While this plane is clearly defined on an ideally flat and chemically homogeneous surface, whose Stern layer is within 1 nm,^[^ ^55^ ^]^ a much less clear structure is seen at a surface biofunctionalized with a protein layer,^[^ ^50^, ^56^ ^]^ such as the Au/anti‐IgM here investigated. It has been already assessed that physisorbed anti‐IgM macromolecules form a very compact and adherent monolayer of proteins on the Au surface.^[^ ^8^ ^]^ Considering that the protein layer is tightly attached to the gold surface and that it is not stripped away by the streaming current, it can be assumed that in the case under study, the actual share plane limit corresponds to the surface of the anti‐IgM protein layer. This is the exact surface inspected by KPFM and by EGOFET. Under this assumption, the ζ‐potential measures the charge associated with the Au/anti‐IgM biolayer, in an *i*_s_ of 10 mm and a pH = 5.5. The ζ‐potentials could not be measured at a lower *i*_s_ (like in deionized water) like for the other potentiometric investigations, as a too‐low streaming current would have flowed. At this higher *i*_s_, λ_D_ goes down to ≈5 – 7 nm so it is smaller by a factor of 20 – 25 than λ_D_ present in the EGOFETs measurements. The protein's charges are, hence, much more screened, and this accounts for the much lower ζ‐potential measured as compared to the corresponding *V*_T_ and SPD. Even at this higher *i*_s_, however, the anti‐IgM layer is still within λ_D_ and hence it can be assumed that the charge measured is associated with a still, partially unscreened protein layer.

To unify the EGOFET data and the ζ‐potential ones, a quantitative estimate of the potential reduction, going from an *i*_s_ of 5·10^−6^ M (EGOFET) to one of 10^−2^ m (ζ‐potentials), can be given considering that the following linear relationship between the ζ‐potential and the molar concentration of ions in the electrolyte solution:^[^ ^57^ ^]^

(8) $$\zeta =a_{0}+a_{1}pC$$

where *pC* is defined as – $log_{10}\sum_{i}n_{i}$, with *n_i_* being the molar concentration of all ionic species in solution. *a_0_* is negligible for a KCl buffer, while *a_1_* parameter depends on temperature, surface modification, and counterion type.^[^ ^58^ ^]^ For biomolecules in KCl buffer, experimental data allow to estimate an *a*_1_ value of ≈45 mV per molar decade of ionic species.^[^ ^59^, ^60^ ^]^ Therefore, a lowering of the measured potential of ≈180 mV can be estimated when using KCl (i_s_ = 10 mm) as a buffer solution instead of water (*i*_s_ = 5 µm). This is in perfect agreement with the data given in Table 1, where a decrease of potential of 197 ± 53 mV is registered between the *V*_T_ and SPD average values before EF‐cycling, which is – (218 ± 53) mV, and the corresponding ζ‐potential value, – (21 ± 6) mV. Hence the difference in the potentiometric values measured with ζ‐potential and EGOFET/KPFM, can be fully accounted for by the different salinity in which the measurements are carried out. To sum up it is possible to state that this section provides a rational unified description of all the SPD (*Φ*_S_/e), *V*_T_, and ζ‐potential values measured that are demonstrated to be comparable within the corresponding errors. This is expected as the three measured potentials (*Φ*_S_/e, *V*_T_, or ζ‐potential) are determined by the same surface charge present on the Au/anti‐IgM surface. Table 1 further evidence that the average values of *Φ*_S_/e, *V*_T_, and ζ‐potential do not significantly change after EF‐cycling.

As the last part of this section, further pertinent information, derived from the ζ‐potential data in Figure 3c, is discussed. From these data, we know that the net charge on the inspected surface (before EF‐cycling) is negative at a pH higher than 3.0 ± 0.1. As we operate at a pH of at least 5.5, we can assume that the surface of the anti‐IgM monolayer on Au is negatively charged as schematically depicted in Figure 3a. A model of the overall dipole moment orientation at the Au/anti‐IgM outmost interface (facing the electrolyte), can be proposed based on this evidence. In the bottom‐left panel of Figure 6, the shift of Φ toward lower values upon anti‐IgM physisorption is accounted for, by assuming that the proteins and their associated overall dipole moments, P_anti‐IgM_, are on average oriented with their negative pole facing the gold surface. As this surface is negatively charged, the negative pole of the anti‐IgM should be repelled. However, this would be the case if the physisorption is driven by electrostatic forces. But these are not likely to be in place as the anti‐IgM physisorption takes place in the high *i*_s_ PBS solution where the charges are largely screened. This is supported by evidence in the literature showing that the driving force for protein physisorption is mainly non‐electrostatic and induced by hydrophobic interactions.^[^ ^61^ ^]^ The KPFM and EGOFET data show that the Au/anti‐IgM film, *Φ*_Au/anti‐IgM_, is lower compared to that of bare gold, *Φ*_Au_, with*Φ* is proportional to |P|. This means that |P_Au_| > |P_anti‐IgM_| and hence an overall dipole moment with its negative pole at the outer surface persist the anti‐IgM electrolyte interface, thus accounting for the ζ‐potential data.

### 2.6 Discussion of the Potentiometric Data Lower Dispersion after EF‐cycling

A noteworthy phenomenon emerges when the Au/anti‐IgM system undergoes cycling in an electric field. As evident from the presented data, the impact of EF‐cycling is threefold: i) there is a noticeable reduction in potentiometric data dispersion (see Δ*ε*/*ε* in Table 1); ii) an increase or decrease of *Φ*_s_/e or *Φ*/e occurs depending on the Au/anti‐IgM sample inspected (Figures 1i and 2f) as well as a less or more negative charge is measured by ζ‐potential (Figure 3d); iii) there are no significant alterations observed in the secondary structure and in the mechanical and morphological properties of the layer.

The first point i) implies that the as‐deposited biolayer holds a peculiar surface potential energy, measured through SPD = *Φ*_S_/e, *V*_T_, or equivalently *Φ*/e, as well as the ζ‐potential. These values describe in a unified manner, the polarization, P_Au/anti‐IgM_ of the system. P_Au/anti‐IgM_ depends on how the 10^11^–10^12^ proteins of the layer, on average, spontaneously physisorb on the Au substrate. The forces governing protein adsorption on a charged surface (Au in this case) stem from a statistical process where an interplay of kinetic and thermodynamic factors leads to the formation of a non‐fully‐ordered ensemble of anti‐IgMs. These proteins can adopt different conformations upon adsorption, with the occurrence of dielectric rearrangements that can bring them out of the native configuration assumed while in a physiological solution.^[^ ^61^ ^]^ The data presented demonstrate that each of the studied pristine as‐deposited Au/anti‐IgM samples has a different P_Au/anti‐IgM_. More relevantly, these values show significant dispersion, with an average error of 33% across the 31 samples analyzed (Table 1). Considering that *Φ*_S_/e, *V*_T_, and the ζ‐potential have been demonstrated to address the overall dipole distribution, we can consider the dispersion relative error, Δ*ε*/*ε*, over all the 31 samples measured. The overall dipole is a result of the distribution of the protein's dipole moment µ contributing to the layer P_anti‐IgM_. However, while the spontaneously physisorbed layer cannot be totally ordered, the proteins are not randomly oriented either, as P_anti‐IgM_ would be zero and no *Φ*_S_/e, *V*_T_, or ζ‐potential shift would be seen upon Au biofunctionalization with anti‐IgM. This means that the anti‐IgM adsorption from a physiological, high *i*_s_ solution, holds a given degree of order, though low. Considering the average dimensions of an antibody and the anti‐IgM film thickness of ≈8 nm, a nearly edge‐on orientation of the proteins is in place. After EF‐cycling the error shift, Δ*ε*/*ε*, marks a reduction of the data dispersion by 61% (Table 1). This means that the EF‐cycling acts as an external stimulus that induces the layers in the different samples to adopt a more similar P_anti‐IgM_ or, in general, a more similar dielectric configuration, which relates to a more alike orientation of the dipoles µ associated with the antibodies. It has been shown that cycling in an electric field can cause effects similar to thermal annealing, as proven with self‐assembled inorganic crystals that are driven into a less defective and overall more ordered configuration with EF‐cycling.^[^ ^15^ ^]^ So cyclic thermal annealing promotes dielectric reorganization, diffusion, and equilibration within the material, leading to a reduction in internal energy and the attainment of a more stable and energetically favorable state.

**Figure 7:**
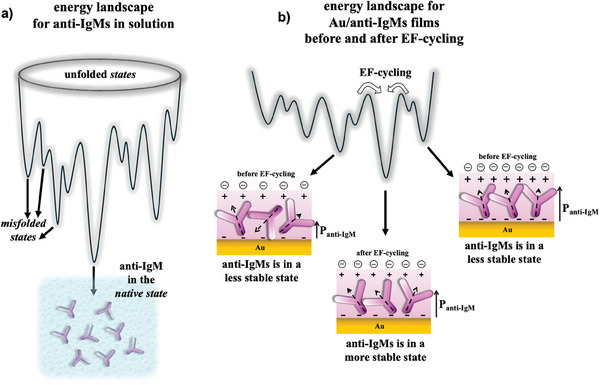
a) Schematic representations of an energy landscape that illustrate the funnel (hypothetical) encompassing pathways for the folding of and anti‐IgM protein in solution. Each path initiates from the highest point of the energy surface where the protein is in its unfolded state and transitions through a sequence of conformations, distinguished by distinct sets of native and nonnative contacts. If the protein takes a path ending in one of the misfolded states it can get trapped and might never end in its functional native state. b) A fictional representation of an energy landscape for an Au/anti‐IgM layer before and after EF cycling. Two less probable and stable states are featured that are energetically different and associated with relative minima. Upon EF‐cycling, they fall into a more probable and stable state.

A similar mechanism for proteins is governed by the complex and irregular nature of the rugged energy surfaces that describe the conformational space of a protein molecule.^[^ ^62^ ^]^ This represents the relationship between the Gibbs free energy of a protein and its conformational space, which encompasses all possible structural configurations the protein can adopt. In **Figure** **7**a a hypothetical representation of a conformational space for an anti‐IgM protein in solution is shown. Protein folding occurs spontaneously and is influenced by hydrophobic interactions, intramolecular hydrogen bonding, Lennard–Jones, and electrostatic interactions. The hydrophobic effect is particularly significant, as it drives the collapse of the hydrophobic regions of a polypeptide chain into the core of the folded protein. In accordance with Anfinsen's dogma, the sequence of amino acids, constituting the primary structure of the polypeptide chain, contains the necessary information to guide the folding process toward its native structure under physiological conditions.^[^ ^63^, ^64^ ^]^

Such complex energy landscapes are characterized by numerous local minima and maxima. The local energy minima represent stable conformations of the protein associated with relatively low energy. These conformations correspond to tertiary folded states or stable intermediates along the folding pathway. The energy landscape also includes transition states, which are higher energy states that the protein must pass through during conformational changes such as folding, unfolding, or transitioning between different structural states.

Proteins can navigate their energy landscapes through multiple pathways, exploring various intermediate states before reaching their final folded or functional form. Interestingly an iterative annealing process carried out at the molecular level has been proposed for proteins to enhance their chances of folding into the correct native state, once they have been trapped into a misfolded state.^[^ ^65^ ^]^ The process involves the interaction with proteins called chaperonins, which bind multiple times to proteins trapped in misfolded conformations, randomly destabilizing their structure, and subsequently releasing them in partially folded states. This process offers proteins numerous chances to explore pathways that lead thermodynamically to the most probable and stable state.

The right panel in Figure 7b depicts the structure of a pristine physisorbed layer of anti‐IgM on Au. During physisorption, misfolding, or partial unfolding is known to occur,^[^ ^61^ ^]^ hence some of these proteins during their physisorption on the gold surface, can become trapped in less stable conformational states associated with one of the several relative minima available. Therefore, an energy landscape for the Au/anti‐IgM system that differs from that of a protein in solution can be envisioned and a hypothetical version of such an energy landscape is also shown in Figure 7b. Eventually, depending on the peculiar deposition/folding route statistically followed, each Au/anti‐IgM layer holds a different *Φ*_S_/e, *V*_T_, or ζ‐potential determining the P_anti‐IgM_. Given a large number of possible relative minima, a rather large dispersion of the P_anti‐IgM_ is seen among the different samples inspected.

EF‐cycling can serve as an external stimulus, acting like an annealing process that enables the anti‐IgM proteins trapped in partially unfolded or misfolded states to reach another minimum characterized by a lower free energy value. This is schematically featured on the left side of Figure 7b. Here an energy landscape for an Au/anti‐IgM layer before and after EF‐cycling is shown. Specifically, two less probable and stable states are represented that are energetically different and associated with relative minima. Upon EF‐cycling, they fall into a more probable and stable state. In fact, the pathways within this landscape can lead to the formation of a layer where the proteins ultimately reach a more probable conformational state. It can be debated that, in this case as well, the genetic encoding of the proteins plays a role, alongside protein‐protein interactions and interactions with the substrate, in driving toward a structure of the anti‐IgM layer that is more probable and stable. The energy level of this conformational state of the film is depicted in Figure 6 with lines and notations in magenta color code.

Regarding the second point ii), a more negative ζ‐potential (Figure 3b) is measured which relates to a higher isoelectric point, associated with a less negative surface (Figure 3d). This feature, also, is sample‐dependent and in this case, it is consistent with a shift toward higher c and P_anti‐IgM_. These are average behaviors, hence, depending on the samples they can shift in one direction or in the other converging anyhow toward a more stable configuration.

Finally, the third point iii) deals with the absence of variation upon EF‐cycling in the PM‐IRRAS spectra which suggests that the secondary structure of the proteins is not involved in the process. Also, the nano‐mechanical properties are unaffected by the EF‐cycling. These properties are generally affected when a rather large conformational change occurs with the protein showing a pronounced hydrophobic character.^[^ ^66^ ^]^

## 3 Conclusions

Investigating the dielectric properties of proteins forming a biolayer is crucial to understanding important aspects connected with biolayer stability. Physisorption of antibodies onto surfaces is convenient and suitable for several applications including bioelectronic sensors, despite the prevailing belief in covalently bound capturing antibodies arranged in ordered structures. In this study, we analyze the effect of cycling in an electric field of an anti‐IgM layer physisorbed on Au by means of KPFM, EGOFETs, and ζ‐potential techniques as well as PM‐IRRAS and F–D spectroscopy to investigate the nanomechanical properties. The anti‐IgM physisorption lowers the Au substrate work function and an overall less negative charge is found on the surface of the Au/protein layer.

During the anti‐IgM physisorption, which lowers the Au work function *Φ* and the P modulus, partial unfolding is known to occur. Hence, during the process, some anti‐IgMs are trapped in less probable states associated with relative minima in the conformational free‐energy landscape. EF‐cycling is deemed to act as an annealing process, bringing the proteins toward a more statistically probable and hence stable, conformational electronic state. The EF‐cycling‐induced dielectric rearrangement, not impacting the infrared and mechanical properties, is associated with a change only in the tertiary structure. Interestingly, an energy landscape of conformational states for the Au/anti‐IgM system can be envisioned that drives the layer toward a more stable state.

## 4 Experimental Section

### Materials

Polyclonal anti‐human immunoglobulin M (anti‐IgM), hydrogen peroxide 30% (w/w), sulfuric acid 96% VLSI grade, phosphate‐buffered saline (pH 7.4, ionic strength 162 mm) were purchased from Sigma‐Aldrich. AZ5214 E photoresist, AZ 726 MIF developer, and AZ100 Remover purchased from Microchemicals are used for UV photolithography and lift‐off process. Poly(3‐hexylthiophene‐2,5‐diyl), P3HT, regioregularity > 99%, with an average molecular weight of 17.5 kDa (g mol^−1^), was purchased from Sigma‐Aldrich and used as field‐effect channel material. All the chemicals are used as received, without an extra purification process.

### Samples Preparation

Gold electrodes used in all the experiments are obtained by electron beam evaporation of a 50 nm thick Au film deposited on top of a 5 nm‐thick Ti adhesion‐promoting layer. The Ti/Au layers are deposited either on Si/SiO_2_ wafers for KPFM measurements or on glass slides (2.5 × 7 cm^2^) for PM‐IRRAS experiments. The substrates are cleaned with a piranha solution H_2_SO_4:_ H_2_O_2_ = 3: 1 bath, rinsed in abundant deionized water and dried with nitrogen. Before the biofunctionalization, the gold‐plated samples undergo a 10‐minute treatment in a UV‐ozone cleaner. This treatment was skipped on the samples inspected by KPFM because, during the physisorption, a part of the sample was masked. If the ozone cleaning was performed before applying the mask, the Au surface became much more reactive at the expense of the chemical inertness necessary for gold to be a good reference. If this cleaning was performed after applying the mask, the treatment partially modifies the surface of the film and worsens the adhesion to the substrate, and there was no longer a sharp interface.

### Physisorption of the Anti‐IgM Layer

The biofunctionalization results in a physisorbed layer of anti‐IgM proteins with a density of 10^4^ proteins µm^−2^.^[^ ^37^ ^]^ This was carried out by incubating the cleaned gold‐plated substrate in a solution of 50 µg mL^−1^ anti‐IgM in PBS for 2 hours, at 21 °C. The samples are then extensively rinsed sequentially in PBS and water and dried by spinning in the air before use to remove the non‐tightly physisorbed proteins. A very stable layer remains on the sample.^[^ ^8^ ^]^ The samples investigated by KPFM are patterned by masking a part of the gold substrate to selectively deposit the biolayer onto the exposed area. Upon removal of the mask, an interface between the Au and the anti‐IgM region was formed. Further details can be found in the referenced literature.

### The EF‐cycling Protocol

The biofunctionalized electrodes undergo a cycling process in an electric field (EF‐cycling). This was carried out by dipping the sample in a custom cell filled with deionized water (HPLC‐grade, pH ≈ 5.5, *i*_s_ ≈ 5 µm) and equipped with a gold counter electrode. These two electrodes were set at a given distance with a spacer of 1 cm. The sample and the counter electrode were connected to a potentiostat, and the potential was swept back and forth in the [0.1−0.5 V] range in steps of 100 mV s^−1^ for 20 times consecutively. Before and after the EF‐cycling, the sample was inspected.

### Representation of the Dataset Distributions

All single data points were plotted as the average over at least five replicates and the error bars, in the point values plots, mark one standard deviation. The datasets of pristine and EF‐cycled anti‐IgM samples collected through KPFM, EGOFET, ζ‐potential, and AFM Force‐Distance spectroscopy measurements were analyzed using the boxplots, to show the measured variables center, spread, asymmetry and to spot the presence of outliers.^[^ ^67^ ^]^ The boxplot displays the samples’ median as a line inside each box, showing the center of the distribution. The average value was given as a star symbol. The average value was, customarily, calculated by summing all the values in a distribution and dividing the total by the number of observations. It represents the central tendency by providing a numerical average of the data points. In contrast, the median was the middle value when all the data points were arranged in ascending or descending order. Each number with the same value was repeated according to its numerosity. Hence, the median was the value that divides the data set, into two equal parts. For distributions with outliers or skewed data, the median was generally a more robust measure of central tendency, as it was less influenced by extreme values, whereas the mean was more sensitive to such outliers. The top and bottom edges of each box were the upper and lower quartiles, respectively. Specifically, 25% of the data fall below the lower quartile value while 75% of the data fall below the upper quartile value. The boxplot shaded area represents the interquartile range (IQR), being the spreading of 50% of the samples. The IQR evaluates the data spreading on a narrower range than the standard deviation, which measures the spreading of 68.2% of the data. The error bars indicate the lowest and highest data points in the dataset excluding any outliers. In the boxplots, the error bars were computed as 1.5 times the IQR. The outliers, falling outside the boundary of the error bars, were plotted as hollow circles.

### Kelvin Probe Force Microscopy (KPFM)

The KPFM measurements were performed using an NT‐MDT system NTEGRA Spectra (Moscow, Russia), in the conventional dual pass amplitude modulation mode, with as‐received Pt/Ir‐coated (Nanosensors, PPP‐EFM) cantilevers, with nominal mechanical resonant frequency and spring constant of 75 kHz and 2.8 N m^−1^, respectively. Each scan line was recorded in two consecutive passes. During the first pass in semi‐contact mode, morphology and phase images were recorded. During the second pass, the surface potential was measured, with the tip lifted 250 nm above the sample surface. This mode limits the tip‐surface interactions to long‐range electrostatic forces,^[^ ^2^, ^68^ ^]^ thus minimizing the influence of topographical features on the measurement of the potential. The KPFM measurements were performed via the application of a DC and an AC voltage to the tip. V_AC_ generates oscillating electrical forces between the tip and sample surface, while V_DC_ nullifies the oscillating electrical forces originating from the contact potential difference between the tip and sample surface. The output signal of a lock‐in amplifier was used to measure the contact potential difference. This signal was recorded for each point on the sample surface, allowing the mapping of surface potential across the sampled area. KPFM allows measuring changes in the surface potential of a gold surface before, Φ_*S*, *Au*_ and after, Φ_*S*,*biolayer*_, the physisorption of a patterned layer of anti‐IgM on Au. The analytical parameter used was the surface potential difference, *SPD* ≡ (Φ_*S*,*Au*_ − Φ_*S*,*biolayer*_)/*e*, so that the gold portion of the sample serves as internal reference. Areas of 90 × 90 µm^2^ across the Au/anti‐IgM interface were scanned, before and after the cycling treatment, always at the same scanning location, over 10 replicate samples. Before the inspection, the samples were thoroughly rinsed in deionized water and dried by spinning at 3000 rpm in air for 30 seconds. All measurements were performed in air at room temperature (22 °C) and the images were processed using the Image Analysis software.

### Electrolyte Gated Organic Field‐Effect Transistors (EGOFETs)

The EGOFETs fabrication starts with the UV lithography definition of source (S) and drain (D) interdigitated electrodes. The spacing between the S – D fingers (channel length, L) and the total finger length (channel width, W) were *L* = 5 µm and *W* = 10.500 µm, respectively. A P3HT solution (4 mg mL^−1^ in chlorobenzene) filtered through a 0.2 µm filter was spin‐coated (2000 rpm, 20 s) and annealed at 90 °C for 15 min. A polyurethane well was glued on the substrate to include the interdigitated channel area and filled with 300 µL of deionized water (HPLC‐grade) acting as a gating medium. Two circular electrodes (0.25 cm^2^) serving as reference and gate (G) were fabricated as Ti/Au‐coating on Si/SiO_2_ substrates. The gate electrode was biofunctionalized with the anti‐IgM capturing layer. Upon source‐drain (*V*_D_) and gate‐source (*V*_GS_ bias, the resulting S–D current was: $I_{D}=\frac{W\cdot \mu_{FET}\cdot C_{i}}{2L}{\left(V_{GS}-V_{T}\right)}^{2}\;at\;|V_{D}\left|>\right|V_{D}^{sat}|$, with *C_i_* being the gating capacitance, *V*_T_ being the transistor threshold voltage, and µ_FET_ being the field‐effect mobility.^[^ ^16^, ^23^, ^69^ ^]^ The $\sqrt{I_{D}}$ versus *V*_G_ plot was customary used to graphically extract the threshold voltage *V*_T_ value, as the intercept with the *V*_G_.^[^ ^52^, ^70^ ^]^ *I*_D_ was stabilized by measuring the transfer characteristics (*I*_D_ vs *V*_G_ with *V*_G_ ranging from 0 to – 0.5 V at a fixed *V*_D_ of – 0.4 V) every half an hour until the *I*_D_ transient current drift measured at *V*_G_ = – 0.5 V and *V*_D_ = – 0.4 V, reduces to 1% per hour.^[^ ^31^ ^]^ The gate in this case was a bare, non‐biofunctionalized, gold‐plated reference electrode.

### Solid Surface ζ‐Potential of the Physisorbed Anti‐IgM layer

The zeta (ζ)‐potential of the pristine and cycled physisorbed anti‐IgM layers was measured with a SurPASS 3 apparatus, by registering the current generated when streaming an electrolyte solution through an adjustable gap system.^[^ ^36^ ^]^ Here, a SurPASS 3 adjustable clamping cell was used for the non‐destructive ζ‐potential analysis of rigid glass samples (17.5 cm^2^ wide) covered by a 50 nm thick gold layer coated with physisorbed antibodies, versus a reusable polyvinylidene difluoride (PVDF) reference surface. For the measurement, the anti‐IgM coated glass was secured on the sample holders and inserted into the cell, with anti‐IgM coating facing the PVDF reference surface, and a gap of ≈100 µm was adjusted between the two surfaces. A buffered KCl electrolyte solution, with *i_s_* of 10 mm and pH 5.5, fills the gap and serves as an ionic conductor in the ζ‐potential measurements. Before starting the measurement, the sample and the cell were thoroughly rinsed with the KCl electrolyte to remove any air from the measuring cell. The ζ‐potential data were acquired by settling a pressure ramp ranging from 400 mbar to 200 mbar and registering three subsequent measurement cycles.

### Polarization Modulation Infrared Reflection‐Absorption Spectroscopy

PM‐IRRAS spectra were recorded using a Nicolet iS50 Fourier transform infrared (FT‐IR) spectrometer equipped with a wire‐grid linear polarizer, a photoelastic modulator (PEM), a sample holder, and a liquid nitrogen‐cooled mercury‐cadmium‐telluride (MCT) detector. The sample was illuminated with mid‐IR radiation at a grazing incidence angle of 82°. The PEM modulates the polarization state of incident radiation between s‐ and p‐polarization (respectively with the electric field parallel and perpendicular to the sample surface) at a frequency of 100 kHz. The apparatus was constantly purged, and the temperature was kept in the 22–24 °C range. For each sample, two PM‐IRRAS spectra were recorded, changing the PEM control unit settings to maximize the signal‐to‐noise ratio (SNR) in the amide I and amide II spectral region (1500–1700 cm^−1^), and in the amide A spectral region (3300 cm^−1^) respectively. Each spectrum was averaged over 1.000 interferograms, with 4 cm^−1^ resolution. The baseline correction on the original PM‐IRRAS signal was calculated in the 1350–1800, 2830–3120, and 3105–3600 cm^−1^ ranges using a least‐square polynomial fitting method. The reflective metallic substrates were Ti/Au‐coated glass slides. Two sets of samples were prepared. The PM‐IRRAS spectra of five samples of the anti‐IgM layer were measured as prepared. Further, five analogous samples were prepared and cycled before PM‐IRRAS measurements. To assess changes in proteins' secondary structure induced by cycling the Amide I band was analyzed. Since the signal‐to‐noise ratio (SNR) in the original PM‐IRRAS spectra was >50, no smoothing was performed. The second derivative spectra were used to identify the main Amide I components. Seven minima bands were identified. Then, fit deconvolution of Amide I was computed using a nonlinear least‐squares method, considering seven Gaussian functions with equal line widths. The wavenumbers of the second derivative minima were used as starting parameters for the fitting Gaussian function peaks. Finally, the relative amount of each secondary structure of the anti‐IgM layer was calculated as the percentage of the total Amide I area for each Gaussian function. Note that the PM‐IRRAS signal on metallic substrates was proportional to the projection of the transition dipole moment along the z‐direction, as depicted in Figure 4a. As protein adsorption on the substrate may induce preferential orientations of the proteins, the peak area must not be interpreted as the real content associated with the corresponding secondary structure but only as its contribution to the final spectrum.

### Force‐Distance Spectroscopy (F–D)

The anti‐IgM samples Young's modulus, deformation, and adhesion force were extracted from F–D curves measured in MilliQ water. The measurements were conducted in contact mode using AFM Bruker NPG‐10 B tips (nominal radius 20 nm; *f*_0_ ≈ 23 kHz; k ≈ 0.12 N m^−1^). The cantilever was first calibrated on a freshly cleaved mica surface in MilliQ water. Then, force curves were collected in 4 × 4 arrays on different areas of a biofunctionalized sample in quintuplicate to characterize the protein layer before and after cycling. Force‐distance curves were analyzed using the AtomicJ software, allowing estimation of parameters such as Young's modulus, deformation, and adhesion force of the surface. For these measurements, a ramp distance of 150 nm, a ramp rate of 10 nm s^−1^, 1000 points per line, and an applied force of 2 nN were adopted.

## Conflict of Interest

The authors declare that they have no conflict of interest.

## Supporting information



Supporting Information

## Data Availability

The data that support the findings of this study are available in the supplementary material of this article.
