# Supplementary material for: Electric Field Cycling of Physisorbed Antibodies Reduces Biolayer Polarization Dispersion
Source: Adv Sci (Weinh). 2024 Nov 8;12(1):2412347. doi: 10.1002/advs.202412347 (PMC11714235; doi:10.1002/advs.202412347)
Supplement: Supplementary file 1 — Supporting Information [file ADVS-12-2412347-s001.pdf]

ADVANCED SCIENCE
Open Access

Supporting Information

for *Adv. Sci.*, DOI 10.1002/advs.202412347

Electric Field Cycling of Physisorbed Antibodies Reduces Biolayer Polarization Dispersion

Cinzia Di Franco, Eleonora Macchia, Michele Catacchio, Mariapia Caputo, Cecilia Scandurra, Lucia Sarcina, Paolo Bollella, Angelo Tricase, Massimo Innocenti, Riccardo Funari, Matteo Piscitelli, Gaetano Scamarcio and Luisa Torsi**

Supporting Information

Electric field cycling of physisorbed antibodies reduces biolayer polarization dispersion

Cinzia Di Franco,^ Eleonora Macchia,^ Michele Catacchio, Mariapia Caputo, Cecilia Scandurra, Lucia Sarcina, Paolo Bollella, Angelo Tricase, Massimo Innocenti, Riccardo Funari, Matteo Piscitelli, Gaetano Scamarcio, and Luisa Torsi**

S1. SPD stability over 24 hours and after washing cycles

Kelvin Probe Force Microscopy was employed to assess the effect of repeated washing steps on the surface potential (SP) of gold and anti-IgM surfaces, as well as the surface potential difference (SPD) between the gold area and the biolayer The data are given in **Figure S1**.

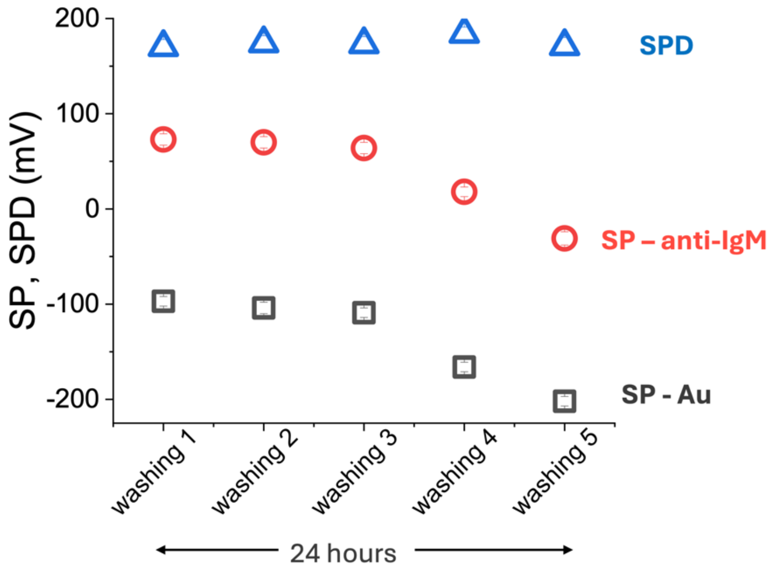

Figure S1. Surface potential (SP) values and surface potential differences (SPD) as a function of repeated washing steps in HPLC water within a 24-hours time-lag. Black squares represent gold (Au) SP, red circles show anti-IgM SP, and blue triangles indicate SPD. Both the biolayer and Au SPs decrease by approximately 100 mV, while SPDs remain nearly constant at (174 ± 6) mV.

Also in this case the Au/anti-IgM sample comprises a very sharp interface between the Au and the anti-IgM portion of each sample investigated. The analysis underwent five washing cycles using HPLC deionized water over a 24-hour period. Each washing step involved submerging the Au/anti-IgM sample in water for 10 minutes, followed by thorough rinsing in deionized water and spin-drying at 3.000 rpm for 30 seconds. After each washing and drying cycle, areas

of 90 × 90 µm² across the Au/anti-IgM interface were imaged, at the same scanning location. The results show that both the gold substrate and the anti-IgM layer experienced a decrease in SP of approximately 100 mV throughout the washing process. Interestingly, despite these changes in individual SP values, the SPD remained remarkably stable at (174 ± 6) mV.

S2. Anti-IgM film thickness

S2.1: Anti-IgM density and biolayer thickness evaluated by Surface Plasmon Resonance (SPR)

The physisorption procedure of anti-IgM is monitored in situ through a Multi-Parameter Surface Plasmon Resonance (MP-SPR) equipment (BioNavis-200 NaviTM instrument in the Kretschmann uconfiguration). [Knoll, W. Interfaces and thin films as seen by bound electromagnetic waves. Annu. Rev. Phys. Chem. 49, 569–638 (1998).] The SPR sensor slides endow a Cr (2 nm)/Au (50 nm) bilayer on a BK7 optical glass. Two laser sources emitting at a wavelength λ = 670 nm are used to study the antibody layer deposition in two areas of the metallic surface (~3 mm spacing). A 0.1 mL flow cell allows the manual-mode injection of the specimen with a syringe onto the 0.4 cm^2 surface inspected. The temperature of the apparatus is set at 21.0 ± 0.1°C and is controlled during the overall deposition. The SPR gold slides are treated with an NH_4OH/H_2O_2 aqueous solution (1:1:5 v/v) at 80-90 °C for 10 min, rinsed with pure water, and cured in a UV-ozone cleaner for 10 min.

Figure S2a shows the SPR sensogram, which displays the angle variation of the plasmon peak minimum *vs.* time. The protocol involves the injection of PBS onto the cleaned gold surface to establish the initial baseline level of the sensogram. After, a solution of anti-IgM (50 µg/ml in PBS) is injected into the flow cell and an increase in the signal is monitored. After 2 h of incubation, a plateau is observed in the sensogram, implying the equilibrium is reached between the uptake of antibodies on the surface and their re-dissolution in the bulk of the buffer solution. To remove any anti-IgM excess, PBS is injected over the modified surface named PBS (1) in **Figure S2a**. A negligible decrease in the signal is observed compared to the plateau level reached after 2 h of antibody physisorption. Also, the layer stability in a non-physiological environment is evaluated by exposing the modified surface to pure water for 20 minutes. The reduction in the sensogram signal is due to the different optical properties of water compared to PBS as a bulk solution. Then PBS (2) is run over the surface to recover the signal in the buffer. A slight decrease of -2% compared to PBS (1) is observed.

Moreover, the MP-SPR collects the whole plasmon peak intensity over the angle range inspected (50.290° - 77.930°). Thus, the thickness of the physisorbed layer can be evaluated by mean of curve-fitting, performed on the plasmon peaks with a multilayer model based on the Fresnel equation using the Winspall 3.02 software. [Winspall 3.02 software Wolfgang Knoll group - Max Planck Institute for Polymer Research, Germany].

Figure S2b shows the plasmon peak variation over the incident laser beam angle acquired before and after the physisorption of anti-IgM on gold. The signal acquired before the antibody deposition (black hollow circle) is compared with the signal gained after the physisorption (green hollow circle). The two fittings performed on the plasmon peaks are reported in **Figure S2b**. These are depicted as a black line for the gold surface before deposition and as a green line for the anti-IgM layer.

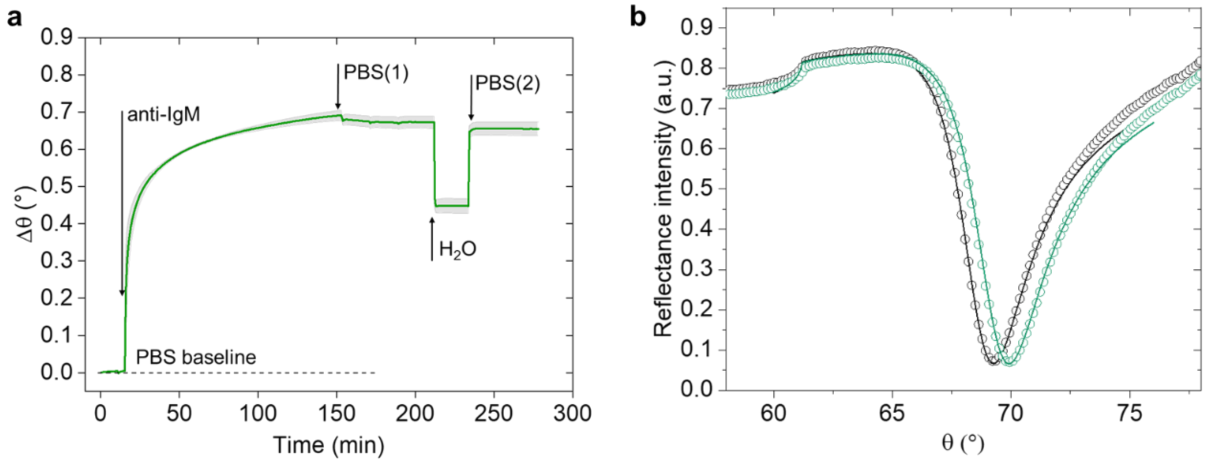

Figure S2: a) SPR sensogram (angle vs. time) showing the real-time deposition of a 50 µg/ml solution of anti-IgM in PBS. The antibodies are injected into the flow cell after acquiring a baseline signal in the PBS buffer. 100 µl of the anti-IgM solution is left in contact with the surface in static mode for 2 hours. Then, the excess is rinsed with the same PBS buffer (1), and a negligible decrease in the signal is observed. The modified surface is exposed to pure water for 20 minutes. The reduction in signal is due to the different optical properties of the bulk solution. PBS buffer (2) is run over the surface to recover the signal.
b) Plasmon peak intensity vs. incidence angle acquired during the physisorption of anti-IgM on gold. The signal acquired before the antibody deposition (black hollow circle) is compared with the signal gained after the physisorption (green hollow circle). The fitting performed on the plasmon peaks is reported for the gold surface before deposition (black line) and the antibody layer deposition (green line). The curve-fitting is performed with a multilayer model based on the Fresnel equation using Winspall 3.02 software.

In **Table S1** the fitting-related data, including the thicknesses and the refractive indices of the anti-IgM layer, are given. The data collected from the two laser sources sampling the two

different areas on the surface are used. An average thickness of 9 ± 2 nm is found for the deposited layer of antibodies.

Table S1: Outputs of the Winspall 3.02 modelling of the data presented in fig.SXXX. The refractive indices, n, or of the real part of the dielectric constant, ε, of the SPR slide (glass and plating layers) and the dielectric constant are provided.

layer	*thickness (nm)*	*real part of the dielectric constant (n or ε)*	*imaginary part of the dielectric constant*
Glass BK7	n.a.*	1.518**	0
Cr	1.9 ± 0.1	2.8 ± 0.2	2.9 ± 0.2
Au	43 ± 1	0.13 ± 0.01	3.79 ± 0.01
anti-IgM	9 ± 2	1.4 ± 0.1	0
PBS	n.a.*	1.33	0

**n.a.:* non applicable; ** value taken from https://refractiveindex.info/ and kept fixed during the modelling

The adlayer of antibodies physisorbed on gold can be further characterized in terms of surface coverage through the experimental data collected. Indeed, the relation between the angle variation calculated from the sensogram (Δθ°) and the surface coverage expressed in ng/cm^2 is defined by the de Feijter's Equation [De Feijter, J. A., Benjamins, J. & Veer, F. A. Ellipsometry as a tool to study the adsorption behavior of synthetic and biopolymers at the air–water interface. Biopolymers 17, 1759–1772 (1978)]:

$$\Gamma = d \cdot (n - n_0) \cdot (dn/dC)^{-1} \qquad \textbf{Eq. S1}$$

where Γ, expressed in $ng \cdot cm^{-2}$, is the surface coverage; d is the thickness of the biolayer deposited on the gold surface; $(n - n_0)$ is the difference between the refractive index of the layer and the one of the bulk medium; and dn/dC is the so-called refractive index increment of the adsorbed biolayer (taken from literature dn/dC ≈ 0.182 $cm^3\ g^{-1}$) [Ball, V. & Ramsden, J. J. Buffer dependence of refractive index increments of protein solutions. Biopolymers 46, 489–492 (1998).]. Also,

$$(n - n_0) = \Delta\theta_{SPR} \cdot k \qquad \textbf{Eq. S2}$$

where k is the sensitivity factor of the instrument which is at λ=670nm 86.3°/RIU ($k=1.16 \cdot 10^{-2}\ deg^{-1}$). [MP-SPR Navi LayerSolver User Manual (2015) BioNavis.

Perlmann et al. (1948). "The specific refractive increment of some purified proteins." J. Am. Chem. Soc., 70, 2719–2724]. Thus, the surface coverage becomes:

$$\boldsymbol{\Gamma} = d \cdot \boldsymbol{\Delta\theta_{SPR}} \cdot k \cdot (dn/dC)^{-1} \qquad \textbf{Eq. S3}$$

If the adlayer deposited is within 100 nm. Here d = 9 ± 2 nm ≈ 10 nm = 10^{-6} cm

$$\boldsymbol{\Gamma} = 10^{-6}\ cm \cdot \boldsymbol{\Delta\theta_{SPR}} \cdot 1.16 \cdot 10^{-2}\ deg^{-1} \cdot (0.182\ cm^3\ g^{-1})^{-1} \sim \mathbf{63.7} \cdot \boldsymbol{\Delta\theta_{SPR}} \qquad \textbf{Eq. S4.}$$

The physisorption of anti-IgM over the gold surface led to a uniform surface coverage of antibodies and the coverage calculated by equation 4 is $\Gamma = (1.711 \pm 0.004) \cdot 10^{11}$. The exposure of the surface to a pure water medium does not significantly compromise the stability of the physisorbed layer. Indeed, the surface coverage calculated after this exposure is $(1.665 \pm 0.004) \cdot 10^{11}$.

S2.2: Anti-IgM thickness evaluated by AFM

The thickness of the anti-IgM layer on the Si/SiO_2 substrate was assessed using a fine needle scratch at the centre of the layer. A sharp tip was used to mechanically remove a strip of the biolayer, taking care not to damage the underlying silicon dioxide surface. This is unlikely to occur as the tip chosen assures a soft contact with the sample surface. The anti-IgM biolayer thickness was determined by measuring the depth of the scratch using semi-contact mode AFM. Line profiles were obtained across the scratch over five 5 x 5 μm^2 areas.

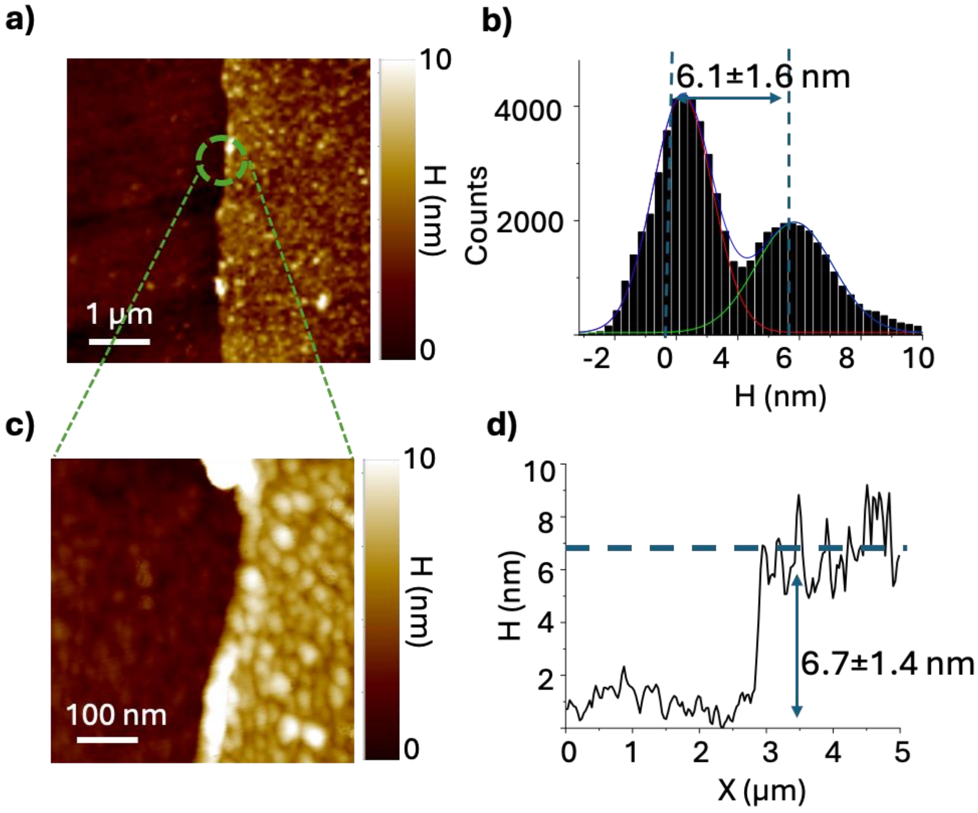

Figure S3: **a)** 5 x 5 μm^2 AFM semi-contact morphology of the anti-IgM on Si/SiO_2, after scratching. **b)** Histogram of the height profiles relevant to the data in panel (a). **c)** High-magnification (500 x 500 nm^2) view of the area highlighted by the green dashed circle in (a). **d)** Representative height profile across the scratch, demonstrating a thickness of 6.7 ± 1.4 nm. Data are averaged over five 5 x 5 μm^2 areas.

Figure S3a displays a representative 5 x 5 μm^2 image of the anti-IgM morphology after the scratch. Ridges formed along the edges of the scratch, protruding above the sample surface, are the result of biolayer displacement during the scratching process. **Figure S3b** shows the Histogram of the height profiles. The distance between the picks is 6.1 ± 1.6 nm. **Figure S3c** shows a higher magnification view of the SiO_2/biolayer interface, clearly revealing both the typical anti-IgM assembly features and the SiO_2 surface. **Figure S3d** features a representative height profile, across the scratch. The height difference between the biolayer and the substrate (used as a reference) was measured, as an average over five 5 x 5 μm^2 areas, to be 6.7 ± 1.4 nm, which clearly corresponds to a monolayer of nearly edge-on anti-IgM antibodies. The two thicknesses are comparable within the errors.
